# Deducting MicroRNA-Mediated Changes Common in Bronchial Epithelial Cells of Asthma and Chronic Obstructive Pulmonary Disease—A Next-Generation Sequencing-Guided Bioinformatic Approach

**DOI:** 10.3390/ijms20030553

**Published:** 2019-01-28

**Authors:** Ming-Ju Tsai, Yu-Chen Tsai, Wei-An Chang, Yi-Shiuan Lin, Pei-Hsun Tsai, Chau-Chyun Sheu, Po-Lin Kuo, Ya-Ling Hsu

**Affiliations:** 1 Division of Pulmonary and Critical Care Medicine, Department of Internal Medicine, Kaohsiung Medical University Hospital, Kaohsiung Medical University, 807 Kaohsiung, Taiwan; SiegfriedTsai@gmail.com (M.-J.T.); 1010362KMUH@gmail.com (Y.-C.T.); 960215kmuh@gmail.com (W.-A.C.); ysirenelin@gmail.com (Y.-S.L.); kanginbobo@gmail.com (P.-H.T.); sheucc@gmail.com (C.-C.S.); 2 Department of Internal Medicine, School of Medicine, College of Medicine, Kaohsiung Medical University, 807 Kaohsiung, Taiwan; 3 Department of Respiratory Therapy, School of Medicine, College of Medicine, Kaohsiung Medical University, 807 Kaohsiung, Taiwan; 4 Graduate Institute of Clinical Medicine, College of Medicine, Kaohsiung Medical University, 807 Kaohsiung, Taiwan; kuopolin@seed.net.tw; 5 Graduate Institute of Medicine, College of Medicine, Kaohsiung Medical University, 807 Kaohsiung, Taiwan

**Keywords:** asthma, COPD, epithelium, bronchial epithelial cells, next-generation sequencing, bioinformatics

## Abstract

Asthma and chronic obstructive pulmonary disease (COPD) are chronic airway inflammatory diseases that share some common features, although these diseases are somewhat different in etiologies, clinical features, and treatment policies. The aim of this study is to investigate the common microRNA-mediated changes in bronchial epithelial cells of asthma and COPD. The microRNA profiles in primary bronchial epithelial cells from asthma (AHBE) and COPD (CHBE) patients and healthy subjects (NHBE) were analyzed with next-generation sequencing (NGS) and the significant microRNA changes common in AHBE and CHBE were extracted. The upregulation of hsa-miR-10a-5p and hsa-miR-146a-5p in both AHBE and CHBE was confirmed with quantitative polymerase chain reaction (qPCR). Using bioinformatic methods, we further identified putative targets of these microRNAs, which were downregulated in both AHBE and CHBE: miR-10a-5p might suppress *BCL2*, *FGFR3*, *FOXO3*, *PDE4A*, *PDE4C,* and *PDE7A*; miR-146a-5p might suppress *BCL2*, *INSR*, *PDE4D*, *PDE7A*, *PDE7B*, and *PDE11A*. We further validated significantly decreased expression levels of *FOXO3* and *PDE7A* in AHBE and CHBE than in NHBE with qPCR. Increased serum miR-146a-5p level was also noted in patients with asthma and COPD as compared with normal control subjects. In summary, our study revealed possible mechanisms mediated by miR-10a-5p and miR-146a-5p in the pathogenesis of both asthma and COPD. The findings might provide a scientific basis for developing novel diagnostic and therapeutic strategies.

## 1. Introduction

Asthma and chronic obstructive pulmonary diseases (COPD) are chronic inflammatory airway diseases characterized by airflow limitation [1,2,3,4,5]. These diseases may cause disabling dyspnea and even death, resulting in a huge socioeconomic burden. Asthma and COPD share some common risk factors and clinical characteristics. Some patients even have some asthma-like clinical features and some COPD-like clinical features, and are sometimes diagnosed as “asthma-COPD overlap” [4,5].

Bronchial epithelial cells play important roles in the pathogenesis of asthma and COPD. These cells are the front-line barrier between the external environment and internal microenvironment in the lungs and are, therefore, susceptible to stimuli from the external environment [6,7]. Increasing evidence shows that bronchial epithelial cells orchestrate the pathobiological mechanisms in the pulmonary microenvironment, contributing to airway remodeling, a major pathophysiological change of chronic airway disease. Many studies have shown that bronchial epithelial cells produce inflammatory cytokines and promote proliferation and migration of bronchial smooth muscle cells [8,9,10].

MicroRNAs (miRNAs, miRs) are small non-coding RNAs involving in post-transcriptional gene repression and are, therefore, important regulators of cellular functions [11]. The microRNAs have been recognized as major regulators in the pathogenesis and disease progression of chronic airway diseases, mainly including asthma and COPD [11,12]. Therefore, microRNAs have great potential to become novel targets for the treatment of asthma and COPD [13].

Although asthma and COPD are usually different in pathophysiological mechanisms, they still share some common changes in gene expression and signal transduction. A study using Ingenuity Pathway Analysis (IPA) has identified some common pathways underlying asthma and COPD, as well as some networks overlapping between asthma and COPD [14]. With more understanding about the common pathophysiological mechanisms underlying both diseases, we may have better opportunity to develop novel treatment strategies that are effective for both diseases. Because microRNAs play important roles in the pathogenesis of asthma and COPD, we believe that the common microRNA-mediated changes in both diseases might be potential targets for both diseases. However, the roles of miRNAs in the pathobiology of asthma and COPD have not been fully understood yet. Therefore, we performed a comprehensive analysis to investigate the microRNA-mediated pathobiological changes, which presented commonly in the bronchial epithelial cells of asthma and COPD.

## 2. Results

### 2.1. Common MicroRNA Changes in the Bronchial Epithelial Cells of Asthma and Chronic Obstructive Pulmonary Diseases (COPD)

From the next-generation sequencing (NGS) results of our previous studies [1,2,3], the differentially expressed microRNAs in diseased (asthma or COPD) bronchial epithelial cells, as compared with normal bronchial epithelial cells, were identified. The microRNAs with changes in the same direction in bronchial epithelial cells of asthma and COPD were selected, which included 7 commonly upregulated and 2 commonly downregulated microRNAs (Table 1).

**Figure 1:**
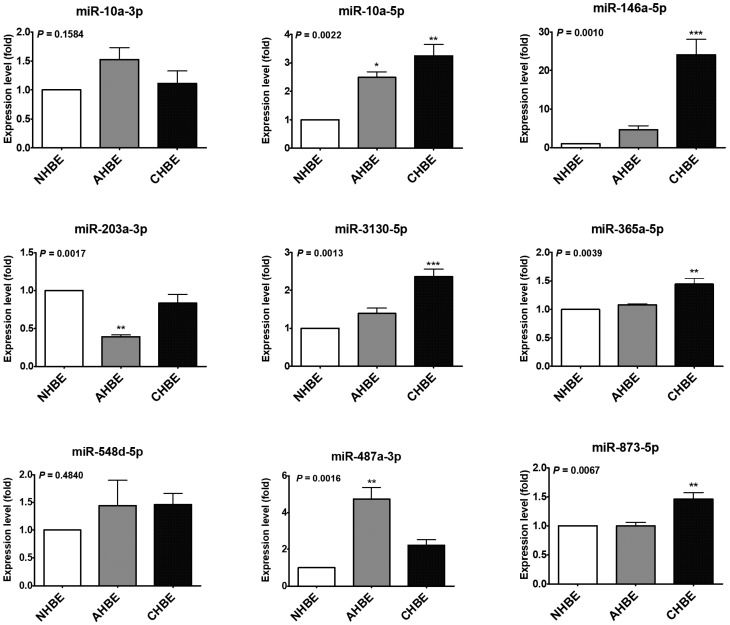
The expression levels of microRNAs in primary human bronchial epithelial cells (HBEs) from a normal subject (NHBE), an asthmatic patient (AHBE), and a patient with chronic obstructive pulmonary disease (CHBE). The expression levels of microRNAs were assessed with quantitative polymerase chain reaction (qPCR). Using the 2^−ΔΔCt^ method, the relative microRNA levels in various cells were calculated. All results were expressed as the mean ± standard error of mean of three independent experiments. The expression levels were compared with analysis of variances followed by Dunnett’s test. * *p* < 0.05, ** *p* < 0.01, *** *p* < 0.001, as compared with NHBE.

To validate the results of NGS, the expression levels of these microRNAs were assessed in another set of primary bronchial epithelial cells (NHBE, AHBE, and CHBE). The expression levels of miR-10a-5p, miR-146a-5p, miR-203a-3p, miR-3130-5p, miR-365a-5p, miR-487a-3p, and miR-873-5p differed significantly in the bronchial epithelial cells from different backgrounds (Figure 1). In these microRNAs, only miR-10a-5p, miR-146a-5p, and miR-487a-3p showed more than two-fold up-regulation in both AHBE and CHBE as compared to the expression level in NHBE. Because the change in miR-487a-3p was paradoxical (downregulation shown in NGS results, but upregulation shown in quantitative reverse transcription polymerase chain reaction (qRT-PCR) results), only miR-10a-5p and miR-146a-5p were taken as the focuses for further investigation.

**Figure 2:**
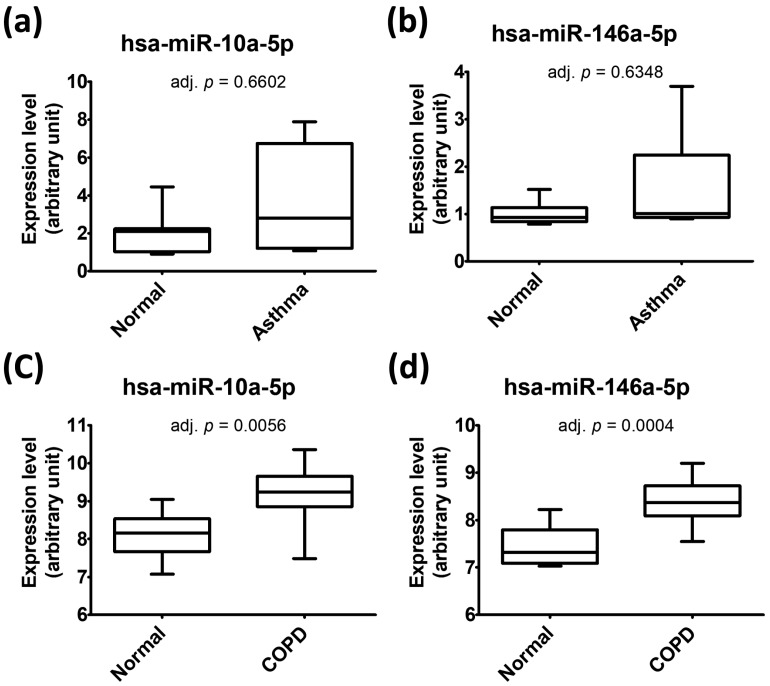
The levels of miR-10a-5p (**a**,**c**) and miR-146a-5p (**b**,**d**) in human bronchial epithelial cells obtained from GSE25230 (**a**,**b**) and GSE38974 (**c**,**d**). The data form Gene Expression Omnibus (GEO) database were analyzed with GEO2R. The *p* values adjusted with method by Benjamini and Hochberg (false discovery rate) (adj. *p*) were shown.

We further used the data from the Gene Expression Omnibus (GEO) database to investigate the levels of both miR-10a-5p and miR-146a-5p in bronchial epithelial cells. Through systematic review of the GEO database, we identified two GEO datasets (GSE25230 [15] and GSE38974 [16]) containing data about both microRNAs in bronchial epithelial cells of asthma/COPD patients and normal subjects. Based on the data in GSE25230, which contained data of seven asthmatic patients and seven normal subjects, the levels of miR-10a-5p and miR-146a-5p in bronchial epithelial cells from asthmatic patients were slightly higher than those from normal subjects (mean log_2_FC: 0.72 and 0.49, respectively) but neither of them reached statistical significance (both adj. *p* value > 0.5) (Figure 2a,b). Because the insignificance might result from small sample size, both microRNAs were still taken as the focuses in our further analyses. GSE38974 was the only GEO dataset (GSE38974) containing data about both microRNAs in bronchial epithelial cells of COPD patients and normal subjects. Based on the data from GSE38974, containing data of 19 COPD patients and 7 normal subjects, the levels of both miR-10a-5p and miR-146a-5p in bronchial epithelial cells from COPD patients were significantly higher than those from normal subjects (mean log_2_FC: 1.04 and 0.95, respectively; both adj. *p* value <0.01) (Figure 2c,d).

### 2.2. Potential Targets of the Upregulated MicroRNAs in the Bronchial Epithelial Cells of Asthma and COPD

**Figure 3:**
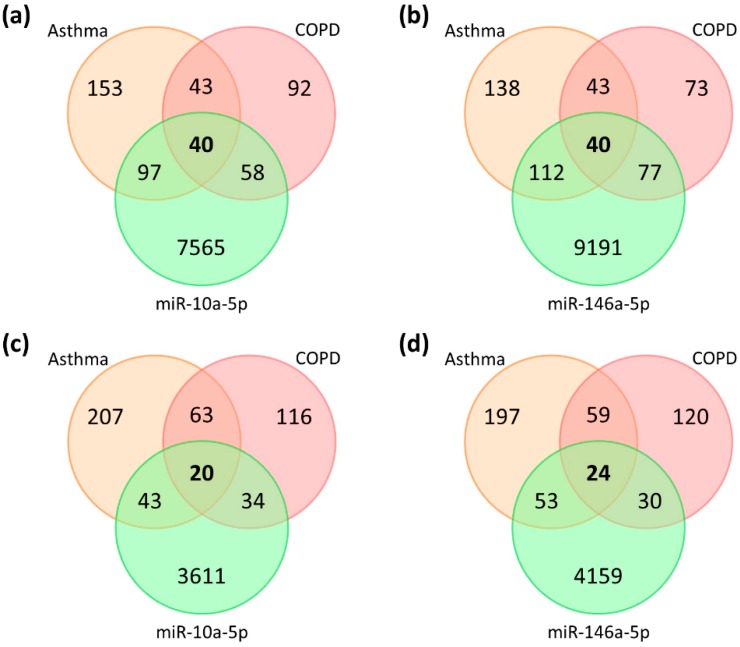
Venn diagrams showing the intersections of asthma-associated molecules, COPD-associated molecules, and the putative targets of miR-10a-5p (**a**,**c**) and miR-146a-5p (**b**,**d**) suggested by at least two (**a**,**b**) or four (**c**,**d**) databases in miRWalk 2.0.

**Figure 4:**
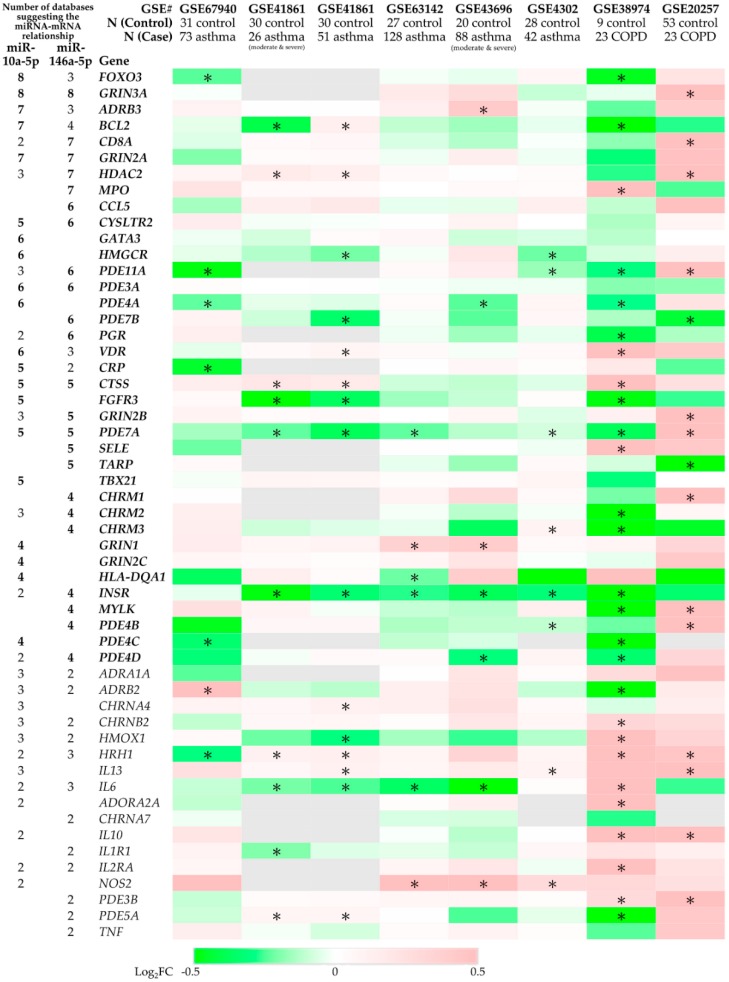
The expression levels of predicted microRNA targets in asthma/chronic obstructive pulmonary disease (COPD) bronchial epithelial cells. The data from the Gene Expression Omnibus (GEO) database were analyzed with GEO2R. The log_2_-fold change (log_2_FC) between two groups (asthma/COPD vs. control) were shown. * The *p* value adjusted with method by Benjamini and Hochberg (false discovery rate) (adj. *p*) < 0.25. The detailed information is presented in Table A2 of Appendix A.

We further tried to predict the potential targets of the remarkably altered microRNAs, which were important in the bronchial epithelial cells of asthma and COPD. Using miRWalk 2.0, we identified 7760 putative targets of miR-10a-5p and 9420 putative targets of miR-146a-5p, which were suggested by at least 2 (out of 12) predicting databases. On the other hand, lists of molecules associated with asthma or COPD were retrieved from the IPA, including 333 asthma-associated molecules and 233 COPD-associated molecules; 83 molecules were associated with both asthma and COPD (Figure 3). Combining the lists of putative microRNA targets and the list of asthma/COPD-associated molecules, we identified 40 asthma-and-COPD-associated genes as putative targets of miR-10a-5p (Figure 3a and Figure 4, Table A1) and 40 asthma-and-COPD-associated genes as putative targets of miR-146a-5p (Figure 3b and Figure 4, Table A1). We further selected the putative targets predicted by at least 4 (out of 12) predicting databases for further analyses (Figure 3c,d and Figure 4, Table A1), including 20 putative targets of miR-10a-5p (*FOXO3*, *GRIN3A*, *ADRB3*, *BCL2*, *GRIN2A*, *CYSLTR2*, *GATA3*, *HMGCR*, *PDE3A*, *PDE4A*, *VDR*, *CRP*, *CTSS*, *FGFR3*, *PDE7A*, *TBX21*, *GRIN1*, *GRIN2C*, *HLA-DQA1*, *PDE4C*) and 24 putative targets of miR-146a-5p (*GRIN3A*, *BCL2*, *CD8A*, *GRIN2A*, *HDAC2*, *MPO*, *CCL5*, *CYSLTR2*, *PDE11A*, *PDE3A*, *PDE7B*, *PGR*, *CTSS*, *GRIN2B*, *PDE7A*, *SELE*, *TARP*, *CHRM1*, *CHRM2*, *CHRM3*, *INSR*, *MYLK*, *PDE4B*, *PDE4D*).

We further used the data from the GEO database to investigate the gene expression levels of bronchial epithelial cells. The significantly down-regulated genes in bronchial epithelial cells from both asthma and COPD patients, as compared with those from normal subjects, were identified (Figure 4, Table A1). These included putative targets of both miR-10a-5p and miR-146a-5p (*BCL2* and *PDE7A*), putative targets of miR-10a-5p (*FGFR3*, *FOXO3*, *PDE4A*, and *PDE4C*), and putative targets of miR-146a-5p (*INSR*, *PDE4D*, *PDE7B*, and *PDE11A*).

**Figure 5:**
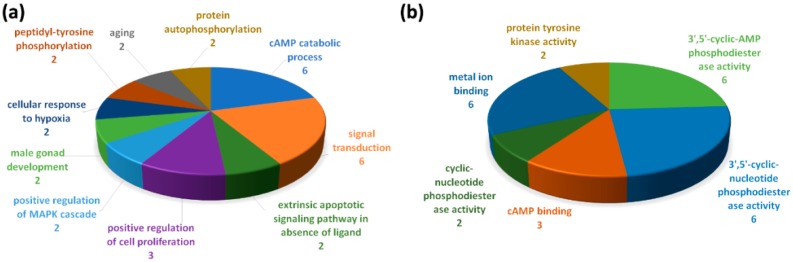
Gene ontology (GO) analyses using the Database for Annotation, Visualization and Integrated Discovery (DAVID) about the putative targets of miR-10a-5p and miR-146a-5p which were significantly down-regulated in bronchial epithelial cells from both asthma and COPD patients. Arabic numerals represent numbers of genes in the (**a**) biological process and (**b**) molecular function.

The 10 putative targets of miR-10a-5p and miR-146a-5p, which were significantly down-regulated genes in bronchial epithelial cells from both asthma and COPD patients, were further analyzed with the Database for Annotation, Visualization and Integrated Discovery (DAVID) (Figure 5). The top 5 biological processes included the adenosine 3′,5′-cyclic monophosphate (cAMP) catabolic process (6 genes), signal transduction (6 genes), extrinsic apoptotic signaling pathway in absence of ligand (2 genes), positive regulation of cell proliferation (3 genes), and positive regulation of the mitogen-activated protein kinase (MAPK) cascade (2 genes). The top 5 molecular functions included 3’,5’-cyclic-AMP phosphodiesterase activity (6 genes), 3’,5’-cyclic-nucleotide phosphodiesterase activity (6 genes), cAMP binding (3 genes), cyclic-nucleotide phosphodiesterase activity (2 genes), metal ion binding (6 genes), and protein tyrosine kinase activity (2 genes).

**Figure 6:**
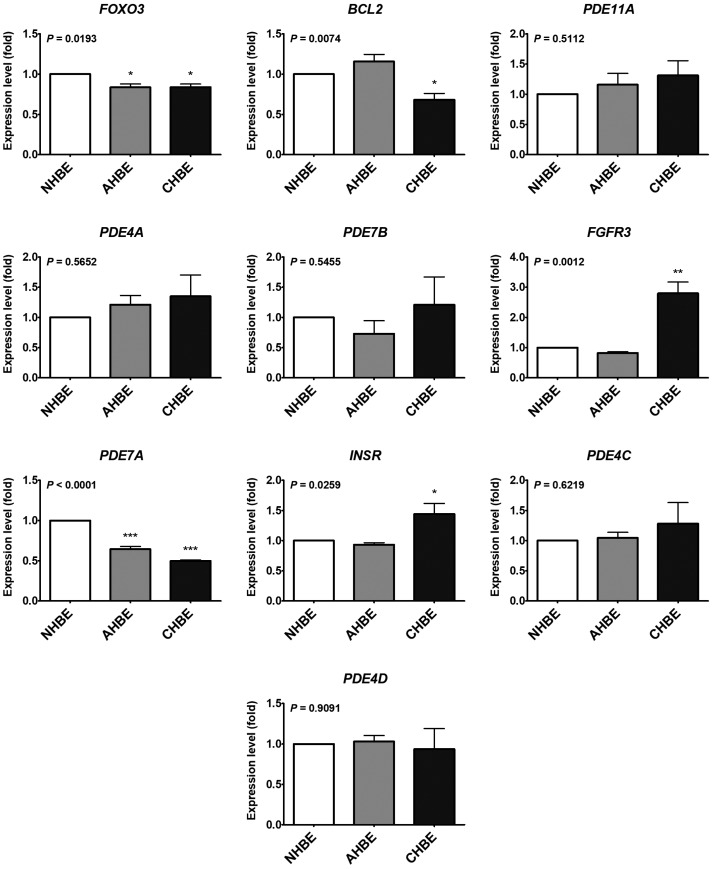
The expression levels of selected genes in primary human bronchial epithelial cells (HBEs) from a normal subject (NHBE), an asthmatic patient (AHBE), and a patient with chronic obstructive pulmonary disease (CHBE). The expression levels of selected genes were assessed with qPCR. Using the 2^−ΔΔCt^ method, the relative mRNA levels in various cells were calculated. All results were expressed as the mean ± standard error of mean of three independent experiments. The expression levels were compared with analysis of variances followed by Dunnett’s test. * *p* < 0.05, ** *p* <0.01, *** *p* < 0.001, as compared with NHBE.

To validate the results of the previous *in silico* analyses using the GEO database, the expression levels of 10 putative targets of miR-10a-5p and/or miR-146a-5p were assessed in the primary bronchial epithelial cells (NHBE, AHBE, and CHBE), which were used in the previous experiment investigating the expression levels of microRNAs. The expression levels of *FOXO3*, *BCL2*, *FGFR3*, *PDE7A*, and *INSR* differed significantly in the bronchial epithelial cells from different background (Figure 6). However, only *FOXO3* and *PDE7A* were significantly decreased in both AHBE and CHBE than in NHBE, while *BCL2* was significantly decreased only in CHBE than in NHBE.

### 2.3. Serum MicroRNA Changes in Asthma and COPD Patients

**Figure 7:**
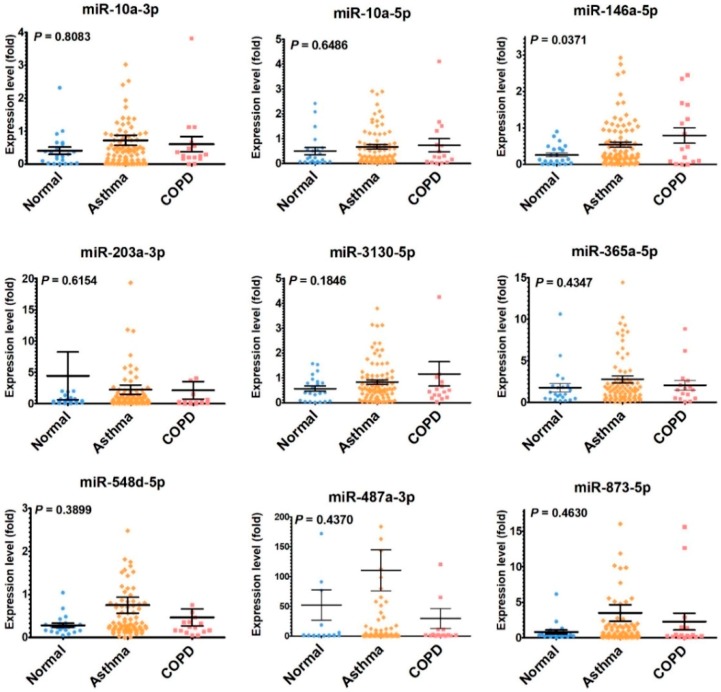
The serum levels of microRNAs in normal subjects, asthmatic patients, and COPD patients. The expression levels of microRNAs were assessed with qPCR. Using the 2^−ΔΔCt^ method, the relative microRNA levels were assessed. The data of subjects were plotted and the mean ± standard error of mean of the group were shown. The expression levels were compared between groups with analysis of variances. Using Dunnett’s post hoc analyses which took the levels of normal subjects as the reference, only the serum miR-146a-5p level was significantly higher in COPD group than that in the normal subjects (*p* = 0.0215), while the serum miR-146a-5p level was not significantly higher in asthma group than in the normal subjects (*p* = 0.1143). For other microRNAs, no significant difference was found with Dunnett’s post hoc analyses.

For clinical correlation, we also analyzed the serum levels of the microRNAs in human subjects. We enrolled 22 normal control subjects, 86 asthma patients and 16 COPD patients (Table 2). The serum microRNA levels were assessed with qPCR-based method. The serum level of miR-146a-5p was significantly higher in asthma and COPD patients as compared with normal control subjects (Figure 7).

## 3. Discussion

In the current study, we have investigated common microRNA changes along with their potential targets in the bronchial epithelial cells of asthma and COPD using NGS and bioinformatic methods. Increased levels of miR-10a-5p and miR-146a-5p were found in bronchial epithelial cells from both asthma and COPD patients. Using bioinformatic methods, we further identified putative targets of these microRNAs, which were downregulated in bronchial epithelial cells from both asthma and COPD: miR-10a-5p might suppress *BCL2*, *FGFR3*, *FOXO3*, *PDE4A*, *PDE4C,* and *PDE7A*; miR-146a-5p might suppress *BCL2*, *INSR*, *PDE4D*, *PDE7A*, *PDE7B*, and *PDE11A*. The putative targets were further validated with primary bronchial epithelial cells, showing significantly decreased levels of *FOXO3* and *PDE7A* in AHBE and CHBE than in NHBE, as well as significantly decreased level of *BCL2* in CHBE than in NHBE. We further confirmed the increased serum miR-146a-5p level in patients with asthma and COPD as compared with normal control subjects.

Few studies to date have shown the roles of miR-10a in pulmonary diseases. Airway smooth muscle cells have abundant miR-10a, which reduces proliferation of the cells via suppressing phosphoinositide 3-kinase (PI3K) pathway [17]. A study using an asthma rat model showed decreased miR-10a promoted proliferation of airway smooth muscle cells, which was associated with increased airway hyper-responsiveness [18]. A mice study revealed the potential role of decreased miR-10a in bleomycin-induced pulmonary fibrosis [19]. Actually, most of the studies about the role of miR-10a in pulmonary diseases focus on its role in lung cancer, but the evidence remains conflicting. Some studies showed up-regulated miR-10a level in non-small cell lung cancer (NSCLC) compared with corresponding normal tissues, which was associated with the proliferation, migration, and invasion of cancer cells [20,21]. However, other studies showed a lower miR-10a level in NSCLC tissues than in adjacent unaffected lung tissue [22]. Another study showed no significant difference in miR-10a level between NSCLC tissue and normal tissue [23]. Interestingly, miR-10a suppressed chemo-resistance to cisplatin and might be used as a potential target for increasing treatment effectiveness [24]. To the best of our knowledge, our study is the first to demonstrate increased miR-10a in bronchial epithelial cells of both asthma and COPD patients as compared with healthy control subjects. Although the mean serum miR-10a-5p level appeared slightly higher in asthma and COPD patients than in normal controls, no statistically significant difference was shown, which might be related to high intra-group variability. This might result from relatively small sample size, and the difference might become statistically significant after the sample size increases. As another explanation, not all microRNAs are secreted from bronchial epithelial cells to the circulation. Therefore, the serum miR-10a-5p level might not directly reflect the level presented in bronchial epithelial cells. Further larger-scale studies are needed to clarify this point.

Being an immune-regulatory microRNA, miR-146a plays an important role in allergy and asthma [25,26]. Several studies have shown the association between miR-146a polymorphism and asthma [27,28,29]. Some studies showed that miR-146a is involved in the production of IgE, promoting the IgE class switch in B cells [30,31]. In line with our finding, some studies showed that children with asthma had significantly higher plasma miR-146a level than healthy controls [32,33]. A recent study even showed that the increased plasma miR-146a level in asthmatics was associated with elevated blood eosinophil count, worse asthma control status, and requirement of higher doses of inhaled corticosteroids [34]. Similarly, increased miR-146a was found in the peripheral blood mononuclear cells from children with allergic rhinitis after allergen-specific immunotherapy [35]. Reduction of miR-146a was found in T cells from patients with severe asthma [36]. However, A549 cells transfected with miR-146a had increased response to glucocorticoids, suggesting that miR-146a might be induced by inflammatory condition and served as a feedback mechanism to limit inflammation [34]. In a study using the ovalbumin-induced asthmatic mice model, miR-146a inhibited the ovalbumin-induced airway hyper-responsiveness and the responses of the group 2 innate lymphoid cells [37]. The enhanced miR-146a expression, shown as increased plasma level, might inhibit proliferation and promote apoptosis of bronchial smooth muscle cells [33]. In contrast, a recent study showed decreased *MIR146A* expression in asthmatic bronchial biopsies as compared to control subjects [38]. Decreased miR-146a-5p expression might be associated with increased CCL20 production from airway smooth muscle cells, which might contribute to enhanced mucus production in asthma [38].

The role of miR-146a in COPD has also been reported in many studies. Single nucleotide polymorphisms of miR-146a were associated with the lung function in COPD smokers [39,40]. In contrast to our study, a study showed significantly decreased serum miR-146a level in COPD patients with acute exacerbation as compared with stable COPD patients and healthy controls, while no significant difference was observed in serum miR-146a level between stable COPD patients and healthy controls [41]. Several studies showed the important role of miR-146a-5p in the aberrant epithelial-fibroblast crosstalk in COPD [42]. IL-1α from airway epithelial cells increased miR-146a-5p expression in primary human lung fibroblasts, which suppressed IL-1α-induced IL-8 production from the fibroblasts; the fibroblasts from COPD patients had less increase in miR-146a-5p expression in response to IL-1α, so the production of IL-8 was less suppressed, which might contribute to chronic inflammation of COPD [42]. Similarly, cytokines induced miR-146a expression to a less extent in COPD fibroblasts than in normal fibroblasts, so the COPD fibroblasts had less suppression of COX-2 activity, resulting in higher PGE_2_ production [43]. In addition, miR-146a might also involve in the networks underlying chronic mucus hypersecretion in COPD [44].

Through the bioinformatic approach, 10 putative targets of miR-10a-5p and/or miR-146a-5p were found. However, we could only validate the significantly decreased expression levels of *FOXO3* and *PDE7A* in both AHBE and CHBE, as well as significantly decreased expression levels of *BCL2* only in CHBE, as compared with the levels in NHBE. *PDE7A* encodes high affinity cAMP-specific 3’,5’-cyclic phosphodiesterase 7A (PDE7A). Previous studies found ubiquitous expression of *PDE7A* in human immune cells, including T cells, eosinophils, neutrophils, and alveolar macrophages, as well as in epithelial cells, vascular smooth muscle cells, and lung fibroblasts [45]. Since PDE7 involved in T cell activation, PDE7 had been considered as a potential therapeutic target for chronic inflammatory diseases including asthma and COPD [46,47,48]. However, a study showed no involvement of PDE7A and PDE7B in the asthmatic mice model of ovalbumin-induced airway inflammation and hyper-reactivity [49]. To the best of our knowledge, no literature has reported the suppressive effect of miR-10a-5p or miR-146a-5p on *PDE7A*. Nevertheless, although PDE7 has been generally considered a pro-inflammatory molecule, its role in bronchial epithelial cells have not been determined, and further study is needed.

The *FOXO3* gene encodes forkhead box O3 (also known as *FoxO3* or *FoxO3a*), which is a transcription factor involved in diverse biological processes. FoxO3 may function as a trigger for apoptosis and is therefore known as a tumor suppressor [50]. Previous studies have shown the association between single nucleotide polymorphism in the *FOXO3* gene and asthma [51,52]. The FoxO3 levels were significantly decreased in the lungs of smokers and COPD patients [53]. As *FoxO3* might disrupt the DNA-binding ability of NF-κB, it might involve the regulation of inflammatory responses in the lungs [53]. FoxO3 might upregulate antioxidants, protecting cells from oxidative stress [53,54]. A recent study using primary bronchial epithelial cells also showed constitutive lower *FoxO3* expression in the cells from COPD patients than in the cells from the controls, and cigarette smoke *extract* (CSE) decreased *FoxO3* expression in the cells from the controls [55]. The decreased FoxO3 level was associated with increased IL-8 and decreased CCL20 expression, which may result in pro-inflammatory responses [55]. Interestingly, a study showed CSE induced SIRT5 to deacetylate *FoxO3*, enhancing nuclear translocation of FoxO3, which mediated the protective effect against CSE-induced apoptosis [54]. Another study also showed the involvement of *FoxO3* in the protective effect of SIRT1 against cigarette smoke-induced oxidative stress [56]. To the best of our knowledge, no literature has reported the suppressive effect of miR-10a-5p on *FOXO3*, whereas the polymorphisms of miR-146a have been associated with altered regulation of *FOXO3* [57]. Nevertheless, based on the current knowledge, we believed that dysregulated miR-10a-5p-*FOXO3* and miR-146a-5p-*FOXO3* in bronchial epithelial cells might be one of the important pathobiological mechanisms underlying both asthma and COPD. 

There were still few limitations in our study. Firstly, the NGS of small RNA was performed with primary bronchial epithelial cells from a single patient in each group (asthma, COPD, and normal subjects). It was difficult to tell whether these findings were related to asthma/COPD, inter-individual variations, or treatment effect. However, using another set of primary bronchial epithelial cells, we confirmed the upregulated changes of miR-10a-5p and miR-146a-5p in patients with asthma or COPD. We also tried to match the race and sex of the sources of primary bronchial epithelial cells to minimize the bias introduced from race and sex. Secondly, the primary bronchial epithelial cells were all from Caucasian patients. Whether our findings could be applied to the Asian population required further investigation. Because we had difficulty in obtaining primary bronchial epithelial cells in our institute, we used serum samples as imperfect substitutes and found increased serum miR-146a-5p levels in patients with asthma and COPD as compared with normal subjects.

## 4. Materials and Methods

### 4.1. Cell Culture

Primary human bronchial epithelial cells from normal control subjects (NHBE), asthma patients (AHBE), and COPD patients (CHBE) were obtained from Lonza Inc. (Walkersville, MD, USA) and cultured as in our previous studies [1,2,3]. In addition to the primary cells used for NGS in our previous studies [1,2,3], we obtained another set of cells, including NHBE (from a 53-year-old male Caucasian non-smoker), AHBE (from a 54-year-old male Caucasian non-smoker with asthma), and CHBE (from a 59-year-old male Caucasian smoker with COPD), for validation of our findings from the analyses of NGS data.

### 4.2. Next-Generation Sequencing (NGS) of Small RNA

The small RNA expression profiles of bronchial epithelial cells were assessed using next-generation sequencing in our previous studies [1,2,3]. The microRNAs with >2-fold changes were considered significantly dysregulated. The significantly dysregulated microRNAs in both asthma and COPD bronchial epithelial cells, with changes in the same direction, were selected for further analyses.

### 4.3. The MicroRNA and mRNA Levels in Bronchial Epithelial Cells

The levels of microRNAs and mRNAs in primary bronchial epithelial cells were assessed with quantitative polymerase chain reaction (qPCR) as in our previous studies [58,59]. In brief, total RNA was extracted from cultured cells using TRIzol Reagents (Thermo Fisher Scientific, Waltham, MA, USA, catalog number: 15596018). The microRNAs and mRNAs were reverse transcribed using the Mir-X miRNA First-Strand Synthesis Kit (TakaRa, Mountain View, CA, USA, catalog number: 638315) and PrimeScript RT reagent Kit (Perfect Real Time) (TaKaRa, catalog number: RR037A), respectively. The microRNA levels were determined using real-time analysis with Fast SYBR Green Master Mix (Thermo Fisher Scientific, catalog number: 4385612) on the QuantStudio 3 Real-Time PCR System (Thermo Fisher Scientific Inc.). The primers used are listed in Table A2. The relative expression levels of the cellular microRNAs and mRNAs were normalized to U6 small nuclear RNA and glyceraldehyde-3-phosphate dehydrogenase (GAPDH), respectively. Using the relative standard curve (2^−ΔΔCt^) method, the relative levels of microRNAs and mRNAs in various cells were calculated [60,61].

### 4.4. Ingenuity Pathway Analysis (IPA) 

Ingenuity^®^ Pathway Analysis (IPA, QIAGEN, Hilden, Germany) [14] is a software integrating many research results (Ingenuity systems, Redwood City, CA, USA), which provides a comprehensive interpretation of extensive experimental data. We obtained asthma- and COPD-associated molecules from IPA.

### 4.5. MicroRNA Target Prediction

The putative targets of microRNAs were predicted using miRWalk 2.0 (http://zmf.umm.uni-heidelberg.de/apps/zmf/mirwalk2/) [62], a comprehensive atlas of predicted and validated miRNA-target interactions. All 12 computational target prediction databases available in miRWalk 2.0 were used for prediction in the current study, including miRWalk, MicroT4, miRanda, miRBridge, miRDB, miRMap, miRNAMap, PICTAR2, PITA, RNA22, RNAhybrid, and TargetScan. Putative microRNA targets suggested by at least 2 (out of 12) predicting databases were extracted and those suggested by at least 4 predicting databases were considered meaningful.

### 4.6. Gene Expression Omnibus (GEO) Database Analysis

The GEO database (https://www.ncbi.nlm.nih.gov/geo/) is a very useful web database, which contains raw gene expression profiles from microarray studies and NGS. Analyzed with GEO2R (https://www.ncbi.nlm.nih.gov/geo/geo2r/), the differentially expressed microRNAs and genes between bronchial epithelial cells from asthma/COPD patients and those from normal control subjects were identified. The *p* values adjusted with the method of Benjamini and Hochberg (false discovery rate) (adj. *p*) [63] and log_2_-fold change (log_2_FC) between two groups (asthma/COPD vs. control) were calculated. The microRNAs and genes with adjusted *p* values <0.25 were considered significantly different between two groups.

The GEO dataset containing the microRNA expression profiles of bronchial epithelial cells from asthmatic patients and control subjects (GSE25230 [15]) and the GEO dataset containing the microRNA expression profiles of bronchial epithelial cells from COPD patients and control subjects (GSE38974 [16]) were selected.

The GEO datasets containing the gene expression profiles of bronchial epithelial cells from asthmatic patients and control subjects (GSE67940 [64], GSE41861, GSE63142 [65], GSE43696 [66], and GSE4302 [67]) and the GEO datasets containing the gene expression profiles of bronchial epithelial cells from COPD patients and control subjects (GSE38974 [16] and GSE20257 [68]) were selected. 

### 4.7. Gene Ontology (GO) Analysis Using Database for Annotation, Visualization and Integrated Discovery (DAVID)

Gene ontology (GO) analysis was performed using DAVID (https://david.ncifcrf.gov/) as in our previous studies [1,2,3]. DAVID is a powerful tool for classification of gene functional [69]. It integrates GO, the biological process, and the Kyoto Encyclopedia of Genes and Genomes (KEGG) pathway. Using DAVID, a list of interesting genes can be classified into clusters of related biological functions, signaling pathways, or diseases by calculating the similarity of global annotation profiles using an agglomeration algorithm method.

### 4.8. Analysis of Serum MicroRNA Levels

Serum was collected from normal healthy subjects, asthma patients, and COPD patients. Approval for the study was obtained from the Institutional Review Board of Kaohsiung Medical University Hospital (KMUHIRB-E(II)-20150139, August 2015). Written informed consent was obtained from all participants in accordance with the Declaration of Helsinki.

Total RNA was extracted from serum using TRIzol LS Reagents (Thermo Fisher Scientific) (3-time of the serum volume). After centrifuging with a mixture of phenol, chloroform, and isoamyl alcohol, the supernatant was incubated with 100% isopropanol in −80°C overnight. After centrifuging at 12,000× *g* for 15 min, the pellet was re-suspended with 75% ethanol. After centrifuging with 75% ethanol for two times, the pellet was dried at 30°C for 30–60 min. Then the RNA pellet was re-suspended in RNase-free water. The cel-miR-39-3p RNA Spike-in template was added using RNA Spike-In Kit, for RT (QIAGEN, Hilden, Germany, catalog number: 339390). The microRNAs were then reverse transcribed and analyzed using the method mentioned above (similarly as in Section 4.3). Using the 2^−ΔΔCt^ method, the microRNAs purified from serum were normalized with cel-miR-39-3p as a spike-in control, and compared with a reference sample (serum kindly donated by a principle investigator, which was used to link and normalize the results from each qPCR plate in order to avoid inter-plate variability).

### 4.9. Statistical Analysis

The levels of microRNAs and mRNAs were compared between groups using analysis of variance (ANOVA), followed by Dunnett’s test. For baseline characteristics of the study population, continuous variables and categorical variables were compared between groups using analysis of variances and chi-square test, respectively. The two-tailed *p*-values less than 0.05 were considered significant. Statistical analyses were performed using the SAS system (version 9.4 for Windows, SAS Institute Inc., Cary, NC, USA). The statistical significance level was set at a two-sided *p* value of <0.05.

## 5. Conclusions

In summary, our study revealed possible mechanisms mediated by miR-10a-5p and miR-146a-5p in the pathogenesis of both asthma and COPD. The aberrant regulations of miR-10a-5p-*FOXO3* and miR-146a-5p-*FOXO3* in bronchial epithelial cells might be important mechanisms underlying both asthma and COPD. The findings might provide a scientific basis for further development of novel diagnostic and therapeutic strategies.

## Figures and Tables

**Table 1:** Common microRNA changes identified from the results of next-generation sequencing (NGS).

microRNA	Expression Level (rpm)		Ratio (AHBE/NHBE)	Expression Level (rpm)		Ratio (CHBE/NHBE)
	AHBE	NHBE *		CHBE	NHBE *	
hsa-miR-10a-3p	6.51	2.58	2.52	2.39	0.14	17.07
hsa-miR-10a-5p	6937.77	2251.48	3.08	52,697.51	24,821.91	2.12
hsa-miR-146a-5p	157.81	53.57	2.95	526.01	223.11	2.36
hsa-miR-203a	2852.52	1184.56	2.41	445.33	187.00	2.38
hsa-miR-3130-5p	1.92	0.65	2.95	19.10	1.67	11.44
hsa-miR-365a-5p	5.18	2.58	2.01	7.50	2.79	2.69
hsa-miR-548d-5p	5.47	2.42	2.26	5.12	2.37	2.16
hsa-miR-487a	1.04	2.74	0.38	2.39	5.86	0.41
hsa-miR-873-5p	1.63	9.84	0.17	1.88	8.65	0.22

* The primary human bronchial epithelial cells (HBEs) used for next-generation sequencing (NGS) included HBE from an asthmatic patient (AHBE), HBE from a patient with chronic obstructive pulmonary disease (CHBE), and HBEs from normal subjects (NHBEs). Because of different timing of the study, different NHBEs were used for the NGS analyses that comparing the expression levels of microRNAs between AHBE and NHBE and between CHBE and NHBE. rpm, read per million.

**Table 2:** Baseline characteristics of the study population.

	Normal Controls	Asthma Patients	COPD Patients	*p* Value *
*n*	22	86	16	
Age	58.67 ± 13.66	46.51 ± 16.27	65.07 ± 12.68	<0.01
Sex				<0.01
Female	16 (73%)	45 (51%)	2 (13%)	
Male	6 (27%)	43 (49%)	14 (88%)	
Body mass index (BMI) (kg/m^2^)	22.77 ± 2.79	25.6 ± 5.26	22.97 ± 4.01	0.01
Obesity (BMI ≥ 27 kg/m^2^)				<0.01
No	21 (95%)	53 (60%)	14 (88%)	
Yes	1 (5%)	35 (40%)	2 (13%)	
Allergic rhinitis				<0.01
No	19 (86%)	27 (31%)	11 (69%)	
Yes	3 (14%)	61 (69%)	5 (31%)	
Smoking history				<0.01
Never smoker	22 (100%)	62 (70%)	2 (13%)	
Current smoker	0 (0%)	16 (18%)	8 (50%)	
Ex-smoker	0 (0%)	10 (11%)	6 (38%)	
Medications				
Oral corticosteroid	0 (0%)	0 (0%)	0 (0%)	
Inhaled corticosteroid	0 (0%)	29 (33%)	5 (31%)	<0.01
Long-acting beta2-agonist	0 (0%)	31 (35%)	7 (44%)	<0.01
Long-acting muscarinic antagonist	0 (0%)	4 (5%)	2 (13%)	0.20
Leukotriene receptor antagonist	0 (0%)	22 (25%)	2 (13%)	0.02
Intranasal steroids	0 (0%)	8 (9%)	0 (0%)	0.16
Theophylline	0 (0%)	2 (2%)	2 (13%)	0.06
Eosinophil percentage (%)	2 ± 1	4 ± 3	3 ± 2	<0.01
Eosinophil count (/μL)	105.67 ± 76.2	299.05 ± 270.23	192.89 ± 116.47	<0.01
Eosinophil cationic protein				0.02
<24 μg/L	22 (100%)	66 (75%)	14 (88%)	
≥24 μg/L	0 (0%)	22 (25%)	2 (13%)	
IgE (IU/mL)	30.93 ± 26.45	485.49 ± 592.8	166.41 ± 165.69	<0.01
FEV_1_ (%predicted)				<0.01
≥80%	22 (100%)	76 (86%)	4 (25%)	
<80%	0 (0%)	12 (14%)	12 (75%)	
FVC (%predicted)				<0.01
≥80%	22 (100%)	74 (84%)	8 (50%)	
<80%	0 (0%)	14 (16%)	8 (50%)	

Abbreviation: FEV_1_, forced expiratory volume in the first minute; FVC, forced vital capacity. * Continuous variables and categorical variables were compared between groups using analysis of variances and Chi-square test, respectively.

## Appendix A

**Table A1:** The expression levels of predicted microRNA targets in asthma/chronic obstructive pulmonary disease (COPD) bronchial epithelial cells.

Number of Databases Suggesting the microRNA-mRNA Relationship		Source	GSE67940		GSE41861		GSE41861		GSE63142		GSE43696		GSE4302		GSE38974		GSE20257	
		*n* (Control)	31 control		30 control		30 control		27 control		20 control		28 control		9 control		53 control	
		*n* (Case)	73 asthma		26 asthma (moderate & severe)		51 asthma		128 asthma		88 asthma (moderate & severe)		42 asthma		23 COPD		23 COPD	
miR-10a-5p	miR-146a-5p	Gene	*adj. p*	log_2_FC	*adj. p*	log_2_FC	*adj. p*	log_2_FC	*adj. p*	log_2_FC	*adj. p*	log_2_FC	*adj. p*	log_2_FC	*adj. p*	log_2_FC	*adj. p*	log_2_FC
8	3	*FOXO3*	0.04	−0.24					0.85	−0.02	0.84	−0.04	0.84	0.07	0.06	−0.47	0.46	0.22
8	8	*GRIN3A*	0.98	−0.01					0.34	0.18	0.41	0.27	0.39	−0.10	0.93	−0.04	0.03	0.70
7	3	*ADRB3*	0.66	0.10	0.93	0.01	0.90	0.01	0.39	0.13	0.12	0.42	0.87	−0.02	0.36	−0.23	0.25	0.40
7	4	*BCL2*	0.92	−0.05	0.01	−0.41	0.01	0.13	0.29	−0.11	0.51	−0.16	0.60	−0.04	0.06	−0.51	0.49	−0.27
2	7	*CD8A*	0.96	−0.06	0.88	0.05	0.84	0.06	0.85	−0.07	0.83	−0.13	0.98	−0.01	0.67	−0.18	0.04	0.78
7	7	*GRIN2A*	0.63	−0.20	0.74	0.03	0.51	0.04	0.56	−0.03	0.69	0.14	0.78	−0.03	0.32	−0.32	0.31	0.49
3	7	*HDAC2*	0.41	0.08	0.01	0.17	0.02	0.13	0.67	0.04	0.97	0.01	0.88	0.03	0.43	−0.27	0.06	0.54
	7	*MPO*	0.62	0.22	0.58	0.04	0.30	0.05	0.97	0.00	0.91	0.05	0.57	0.03	0.16	0.55	0.63	−0.25
	6	*CCL5*	0.89	−0.15	0.63	0.15	0.47	0.19	0.95	−0.04	0.96	−0.05	0.45	0.13	0.80	−0.11	0.27	0.50
5	6	*CYSLTR2*	0.51	0.16	0.84	−0.02	0.83	−0.01	0.94	0.01	0.83	0.10	1.00	0.00	0.62	−0.14	0.87	0.08
6		*GATA3*	0.94	−0.03	0.58	−0.08	0.88	0.03	0.74	0.06	0.91	−0.09	0.49	−0.07	0.71	−0.12	1.00	0.00
6		*HMGCR*	0.86	−0.05	0.28	−0.13	0.03	−0.21	0.93	−0.02	0.48	0.20	0.11	−0.22	0.78	−0.08	0.69	0.14
3	6	*PDE11A*	0.19	−0.48					0.53	0.04	0.57	0.16	0.10	−0.17	0.23	−0.30	0.07	0.69
6	6	*PDE3A*	0.86	0.02	0.98	0.00	0.87	0.01	0.96	−0.01	0.95	−0.03	0.69	−0.03	0.43	−0.19	0.76	−0.18
6		*PDE4A*	0.01	−0.23	0.57	−0.05	0.40	−0.06	0.73	0.03	0.08	−0.24	0.45	0.07	0.05	−0.28	0.46	0.23
	6	*PDE7B*	0.87	0.09	0.28	−0.09	0.00	−0.35	0.82	−0.02	0.28	−0.24	0.25	0.06	0.71	−0.14	0.20	−0.44
2	6	*PGR*	0.52	0.13					0.66	−0.03	0.59	−0.16	0.57	−0.04	0.05	−0.40	0.80	−0.14
6	3	*VDR*	0.81	−0.05	0.42	0.05	0.08	0.08	0.77	0.05	0.95	−0.02	0.55	0.04	0.05	0.50	0.29	0.42
5	2	*CRP*	0.10	−0.45					0.96	0.01	0.93	0.06	0.89	0.02	0.60	0.19	0.61	−0.24
5	5	*CTSS*	0.37	0.15	0.10	0.20	0.10	0.17	0.68	−0.09	0.76	−0.11	0.56	−0.06	0.00	2.14	0.36	0.24
5		*FGFR3*	0.89	0.05	0.00	−0.49	0.01	−0.37	0.48	−0.16	0.85	−0.10	0.80	0.02	0.06	−0.48	0.40	−0.26
3	5	*GRIN2B*	0.72	0.09	0.73	0.03	0.52	0.05	0.98	0.00	0.63	0.07	0.44	−0.06	0.74	0.11	0.03	1.04
5	5	*PDE7A*	0.70	−0.15	0.02	−0.23	0.00	−0.38	0.03	−0.23	0.57	−0.12	0.17	−0.08	0.23	−0.38	0.09	0.66
	5	*SELE*	0.66	−0.22					0.88	0.01	0.98	0.01	0.68	−0.03	0.15	1.34	0.40	0.44
	5	*TARP*	0.94	0.04					0.87	−0.04	0.79	−0.17	0.72	0.05	0.84	−0.08	0.14	−0.60
5		*TBX21*	0.98	−0.02	0.40	0.08	0.37	0.07	0.90	0.02	0.97	0.03	0.45	0.08	0.50	−0.31	1.00	0.00
	4	*CHRM1*	0.98	0.01					0.27	0.10	0.30	0.29	0.55	0.04	0.35	−0.21	0.13	0.66
3	4	*CHRM2*	0.72	0.12					0.82	−0.02	0.73	−0.12	0.79	−0.02	0.00	−1.22	0.90	0.07
	4	*CHRM3*	0.67	0.15	0.29	−0.09	0.28	−0.06	0.41	−0.04	0.46	−0.37	0.17	0.09	0.00	−1.32	0.35	−0.46
4		*GRIN1*	0.72	0.16	0.27	0.07	0.26	0.10	0.04	0.35	0.07	0.40	0.53	0.05	0.73	0.08	0.44	0.32
4		*GRIN2C*	0.83	0.07	0.41	0.05	0.85	0.02	0.51	0.08	0.63	0.21	0.94	−0.01	0.92	−0.04	0.32	0.44
4		*HLA−DQA1*	0.75	−0.37	0.89	0.13	0.98	0.03	0.24	−0.22	0.62	0.40	0.52	−0.55	0.66	0.75	0.50	−0.66
2	4	*INSR*	0.87	−0.04	0.00	−0.48	0.00	−0.33	0.01	−0.27	0.04	−0.38	0.22	−0.32	0.17	−0.56	0.26	−0.34
	4	*MYLK*	0.75	0.24	0.45	0.09	0.93	−0.02	0.43	−0.10	0.68	−0.12	0.49	0.13	0.01	−0.75	0.10	0.59
	4	*PDE4B*	0.26	−0.47	0.54	0.06	0.44	0.06	0.57	−0.14	0.58	−0.11	0.22	−0.11	0.35	−0.22	0.04	0.91
4		*PDE4C*	0.20	−0.34					0.41	−0.11	0.78	−0.07			0.01	−0.54		
2	4	*PDE4D*	0.67	−0.32	0.76	−0.02	0.57	0.05	0.59	0.05	0.17	−0.31	0.42	0.08	0.12	−0.34	0.26	0.33
3	2	*ADRA1A*	0.55	−0.24					0.94	0.01	0.65	0.21	0.67	0.03	0.42	0.28	0.37	0.48
3	2	*ADRB2*	0.00	0.58	0.56	−0.09	0.26	−0.12	0.49	0.13	0.45	0.23	0.72	−0.10	0.04	−0.79	0.53	0.17
3		*CHRNA4*	0.55	0.10	0.49	0.06	0.10	0.11	0.55	0.13	0.46	0.24	0.60	0.05	0.77	−0.07	0.64	0.14
3	2	*CHRNB2*	0.81	−0.11	0.52	0.06	0.31	0.07	0.96	0.02	0.61	0.20	0.45	0.07	0.12	0.32	0.46	0.28
3	2	*HMOX1*	0.79	0.04	0.38	−0.22	0.12	−0.30	0.67	−0.15	0.59	−0.26	0.42	−0.13	0.01	1.32	0.34	0.34
2	3	*HRH1*	0.08	−0.30	0.16	0.11	0.06	0.12	0.38	0.08	0.28	0.34	0.48	0.08	0.00	0.84	0.25	0.45
3		*IL13*	0.69	0.24	0.49	0.06	0.11	0.11	0.37	0.07	0.76	0.17	0.17	0.10	0.55	0.49	0.07	0.71
2	3	*IL6*	0.87	−0.10	0.24	−0.21	0.04	−0.25	0.01	−0.36	0.05	−0.94	0.87	0.02	0.01	2.37	0.61	−0.26
2		*ADORA2A*	0.76	−0.11					0.81	0.02	0.66	0.12			0.00	0.57		
	2	*CHRNA7*	0.92	−0.03					0.50	−0.07	0.84	−0.08			0.32	−0.27		
2		*IL10*	0.57	0.21					0.96	−0.01	0.79	−0.12	0.84	0.01	0.05	0.65	0.21	0.54
	2	*IL1R1*	0.77	0.10	0.17	−0.20	0.73	−0.06	0.73	−0.05	0.76	−0.10	0.56	0.06	0.40	0.22	0.80	0.12
2	2	*IL2RA*	0.93	0.07	NA	NA	NA	NA	0.45	0.08	0.67	0.20	0.75	−0.03	0.01	1.04	0.55	0.23
2		*NOS2*	0.48	0.58					0.01	1.10	0.08	1.15	0.02	0.29	0.50	0.31	0.65	0.24
	2	*PDE3B*	0.71	−0.10	0.70	0.03	0.56	0.04	0.52	0.07	0.87	0.08	0.76	0.02	0.13	0.29	0.02	0.99
	2	*PDE5A*	0.78	−0.09	0.25	0.08	0.17	0.08	0.93	0.01	0.50	−0.25	0.46	−0.05	0.00	−0.79	0.38	0.43
	2	*TNF*	0.55	0.15	0.92	−0.02	0.46	−0.08	0.76	0.03	0.87	0.10	0.90	0.02	0.27	−0.24	0.27	0.43

The data from the Gene Expression Omnibus (GEO) database were analyzed with GEO2R. The *p* values adjusted with method by Benjamini and Hochberg (false discovery rate) (adj. *p*) and log_2_-fold change (Log_2_FC) between two groups (asthma/COPD vs. control) were shown. The results of significant (adj. *p* value <0.25) down-regulation in the asthma/COPD group were highlighted with yellow background; the gene names with at least one significant down-regulation in both asthma and COPD databases were also highlighted with orange background.

**Table A2:** The target sequences of primers used in this study.

microRNA	Primer Sequence
hsa-miR-10a-3p	5′ CAAATTCGTATCTAGGGGAATA 3′
hsa-miR-10a-5p	5′ TACCCTGTAGATCCGAATTTGTG 3′
hsa-miR-146a-5p	5′ TGAGAACTGAATTCCATGGGTT 3′
hsa-miR-203a-3p	5′ GTGAAATGTTTAGGACCACTAG 3′
hsa-miR-3130-5p	5′ TACCCAGTCTCCGGTGCAGCC 3′
hsa-miR-365a-5p	5′ AGGGACTTTTGGGGGCAGATGTG 3′
hsa-miR-548d-5p	5′ AAAAGTAATTGTGGTTTTTGCC 3′
hsa-miR-487a-3p	5′ AATCATACAGGGACATCCAGTT 3′
hsa-miR-873-5p	5′ GCAGGAACTTGTGAGTCTCCT 3′
cel-miR-39-3p	5′ TCACCGGGTGTAAATCAGCTTG 3′
FOXO3 forward	5′ GGACAAACGGCTCACTCTGT 3′
FOXO3 reverse	5′ GCTCTTGCCAGTTCCCTCAT 3′
BCL2 forward	5′ GTGGTGGAGGAGCTCTTCAG 3′
BCL2 reverse	5′ GCCGGTTCAGGTACTCAGTC 3′
FGFR3_H_F2	5′ CCAATGTCTCCGAGCTCGAG 3′
FGFR3_H_R2	5′ TCCTTGTCAGTGGCATCGTC 3′
PDE11A forward	5′ CCACAACTGGAGACATGCCT 3′
PDE11A reverse	5′ TTGTTGGTTCCCCTGTGGTC 3′
INSR forward	5′ TGGCAACATCACCCACTACC 3′
INSR reverse	5′ CCGGCCGAATCCTCATACTC 3′
PDE7A forward	5′ TTGCAGCTGCCACTCATGAT 3′
PDE7A reverse	5′ GTCTCCATTTGTTGCCTGCT 3′
PDE4D forward	5′ ACCGGCCCTTGACTGTTATC 3′
PDE4D reverse	5′ ACTGGACAACATCTGCAGCA 3′
PDE4A forward	5′ CGATTTGGGGTGAAGACCGA 3′
PDE4A reverse	5′ TGTCACCATCGTGTCCACAG 3′
PDE4C forward	5′ CTGACCAGGAGGAGCAACTG 3′
PDE4C reverse	5′ TGACCTTCCAGCATCAGCAG 3′
PDE7B forward	5′ TGTGTTTGCATGCTGGGAGA 3′
PDE7B reverse	5′ TTCCACGAAGCAGCCTTGAT 3′
